# Analysis of the Association Between BNT162b2 mRNA COVID-19 Vaccination and Deaths Within 10 Days After Vaccination Using the Sex Ratio in Japan

**DOI:** 10.7759/cureus.50144

**Published:** 2023-12-07

**Authors:** Yasusi Suzumura

**Affiliations:** 1 Division of Research, YSP Medical Information Laboratory, Aichi, JPN

**Keywords:** vaccine safety, covid-19 vaccines, self-controlled risk interval design, deaths after vaccination, sex ratio

## Abstract

Introduction: The association between coronavirus disease 2019 (COVID-19) vaccinations and deaths after vaccination has been investigated primarily through cohort and self-controlled case series studies. In the present study, the sex ratios of reported deaths were compared by period.

Methods: Descriptive analysis was conducted using data on deaths reported after vaccination with the BNT162b2 mRNA vaccine. The data used were published by the Ministry of Health, Labour and Welfare in Japan. The risk period was defined as within 10 days of vaccination, and the control period was defined as 11 to 180 days after vaccination. Sex ratios were calculated for all-cause deaths and each outcome by dividing the number of males by that of females and multiplying by 100. Fisher's exact test was performed to analyze the results. Graphs were created to show the number of days from vaccination to death and that of reported deaths.

Results: For all-cause deaths among individuals aged ≥65 years, the sex ratio during the risk period was 92, significantly lower than that during the control period (130) (p=0.0050). Conversely, for all-cause deaths among those aged ≤64 years, the sex ratio during the risk period was 204, significantly higher than that during the control period (111) (p=0.044). Reported deaths were concentrated during the risk period in both groups. Sex ratios by period for each outcome were also examined. However, the differences were not significant across any of the outcomes.

Conclusion: The results indicate that the BNT162b2 mRNA vaccination may influence the occurrence of death during the risk period. In a cohort study in Japan, there was no significant increase in all-cause mortality owing to vaccination. This does not contradict the results of the present study. The results of a cohort study provide support for vaccine safety. However, this does not indicate that vaccine-related deaths are nonexistent; it only indicates that their number is not large enough to make a significant difference. Japan has relief services for adverse health effects that provide financial support to patients. On this occasion, it is difficult to determine whether a postvaccination death is incidental or vaccine-related. A self-controlled risk interval design and a comparison of sex ratios by period may be useful in examining the association between vaccination and deaths after vaccination when a cohort study does not detect a significant difference due to a low mortality rate. The latter approach may be particularly useful for analyzing data with reporting bias. The author believes that this approach may not provide conclusive evidence, but it can offer valuable insights into assessing vaccine safety.

## Introduction

The association between coronavirus disease 2019 (COVID-19) vaccinations and deaths after vaccination has been investigated primarily through cohort studies and self-controlled case series studies (SCCSs). These studies showed no significant increase in all-cause mortality due to vaccination [1-3]. In contrast, Yamashita et al. reported in 2022 time-dependent changes in the sex ratio of deaths after the BNT162b2 mRNA vaccination, a COVID-19 vaccination, in Japan. Specifically, there was a significant difference in the sex ratio between the period within 10 days after vaccination and that after 11 days in the reported deaths after vaccination among individuals aged ≥65 years [4]. This indicates that the vaccination may influence the occurrence of death within the initial 10 days after vaccination. This 10-day period was chosen due to the higher frequency of reported deaths during this timeframe [4].

The data on deaths reported after COVID-19 vaccinations were compiled into lists by the Ministry of Health, Labour and Welfare (MHLW) and published on its website [5]. Since the report by Yamashita et al., many deaths suspected to be related to vaccination have been reported to the MHLW in Japan. In the present study, the author analyzed all reported cases, including cases reported after the tabulation by Yamashita et al., using a similar method of comparing sex ratios by period and examined whether significant differences were found. Notably, many deaths after vaccination have also been reported in individuals aged ≤64 years. Therefore, it is important to analyze the sex ratios after the vaccination in those individuals. Yamashita et al. analyzed only reported cases of individuals aged ≥65 years. In the present study, the data of those aged ≤64 were also analyzed. The author discussed how significant differences in sex ratios should be interpreted and under what conditions this method is useful.

## Materials and methods

Data collection on death reports

Data on deaths were sourced from lists published by the MHLW in Japan, detailing cases reported as deaths after COVID-19 vaccinations [5]. The data provided cases in which physicians suspected that the vaccination was associated with death. Specifically, cases involving only the BNT162b2 mRNA vaccination reported between February 17, 2021, and March 12, 2023, were included. Exclusions were made for patients with unknown age, sex, or number of days from vaccination to death. When multiple causes of death were cited, the primary cause was determined based on physicians' comments. As the data were initially in PDF format, they were converted to Excel files and tabulated using Visual Basic for Applications.

Ethical considerations

The study was exempt from ethical review according to the Ethical Guidelines for Medical and Biological Research Involving Human Subjects in Japan because the author used publicly available data.

Analysis

Descriptive analysis was performed to examine the sex ratio among reported death cases. The analysis was divided into two groups: Group 1 included individuals aged ≥65 years and Group 2 included those aged ≤64 years. Sex ratios were calculated by dividing the number of reported deaths of males by that of females and multiplying by 100. The period from vaccination to death was calculated, and the day of vaccination was defined as Day 1. The risk period was designated as the span within 10 days after vaccination, while the control period ranged from 11 to 180 days after vaccination. Sex ratios were compared for each period and calculated for all-cause deaths and each outcome. The author chose to focus on outcomes that were either deemed significant or were observed in 20 or more cases. The outcomes (International Classification of Diseases, 10th revision) include ischemic heart diseases (I21, I22, I24), myocarditis/pericarditis (I30, I40), cardiac arrhythmias (I44, I47, I49), aortic aneurysm/dissection (I71), heart failure (I50), intracerebral hemorrhage (I61), subarachnoid hemorrhage (I60), cerebral infarction (I63), respiratory failure (J96), interstitial lung diseases (J84), pulmonary embolism (I26), pneumonia (J13, J14, J15, J18), sepsis (A40, A41), anaphylaxis (T886), thrombocytopenia (D69, A938, M311), aspiration pneumonia/asphyxia (J690, W78, W79), marasmus (E41, R54), and unexplained deaths (R99). Graphs were created to show the number of days from vaccination to death and that of reported deaths. Fisher's exact test was performed to analyze the results. The author tested the outcomes in 20 or more cases. The tests were conducted using the R software (version 4.1.1; R Foundation for Statistical Computing, Vienna, Austria). Statistical significance was defined as a two-sided p-value of <0.05. The statistical power of the tests for all-cause deaths was calculated using EZR version 1.55 [6].

## Results

**Figure 1:**
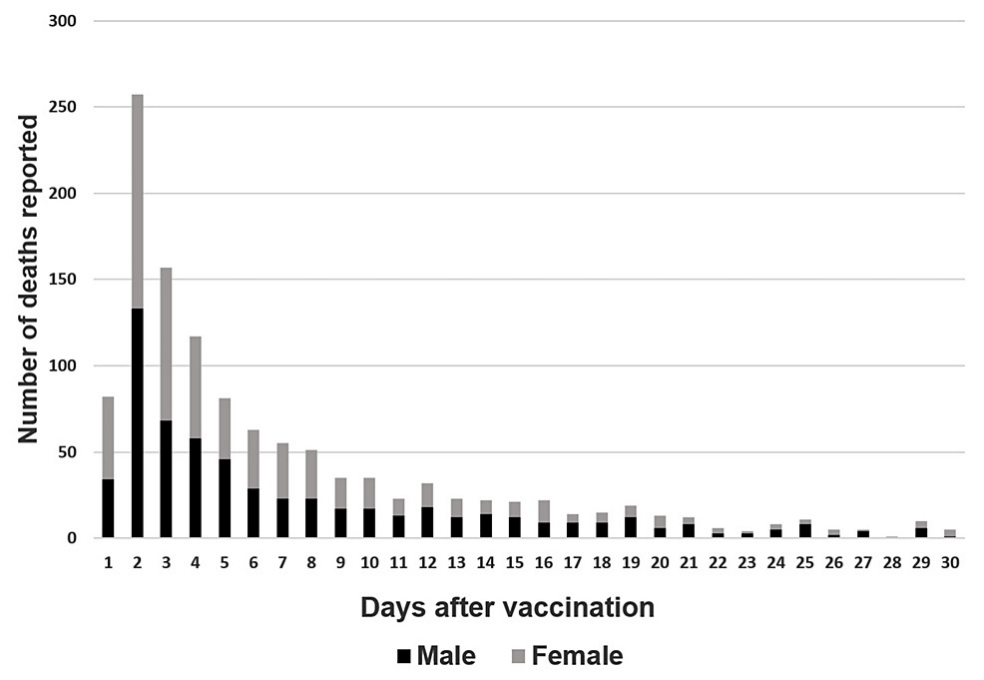
Deaths reported per day after vaccination in individuals aged ≥65 years Graph illustrating the number of days from vaccination to death and death counts in individuals aged ≥65 years. The number of deaths is sorted by sex: male (black) and female (gray).

**Figure 2:**
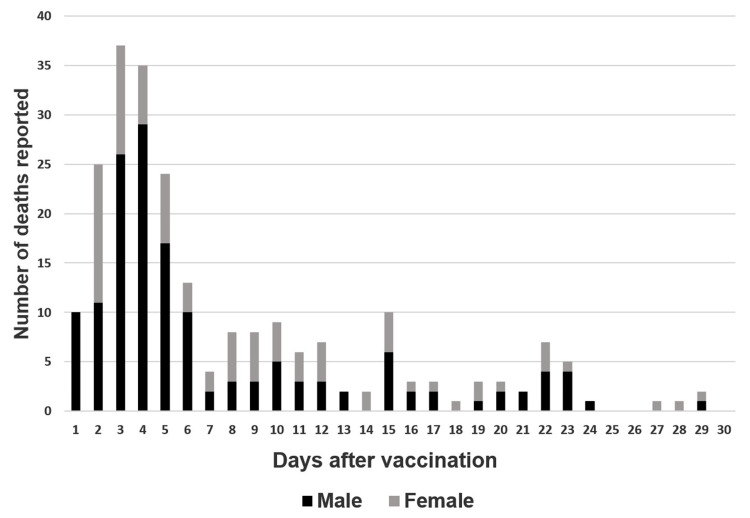
Deaths reported per day after vaccination in individuals aged ≤64 years Graph illustrating the number of days from vaccination to death and death counts reported in individuals aged ≤64 years. The number of deaths is sorted by sex: male (black) and female (gray).

After BNT162b2 mRNA vaccination, 1,311 deaths were identified in Group 1, including 662 males and 649 females, with a mean (standard deviation) age of 82.8 (8.5) years. A total of 247 deaths were identified in Group 2, with 155 males and 92 females, with a mean age of 47.1 (13.7) years. The percentage of reported cases that experienced death within 10 days after vaccination was 71% in Group 1 and 70% in Group 2 (Figures 1, 2).

In Group 1, the sex ratio for all-cause deaths during the risk period was 92, which was significantly lower than the 130 observed during the control period (p=0.0050). Regarding the outcomes in ≥20 cases, the sex ratio during the risk period was lower than that during the control period for conditions including ischemic heart disease, aortic aneurysm/dissection, intracerebral hemorrhage, subarachnoid hemorrhage, cerebral infarction, interstitial lung diseases, pneumonia, aspiration pneumonia/asphyxia, marasmus, and unexplained deaths, whereas no significant differences were observed between the sex ratios by period. For unexplained deaths, autopsies were performed in eight of the 239 cases. The statistical power of the tests for all-cause deaths was 0.79. The sex ratios and p-values for each outcome are summarized in Table 1.

**Table 1:** Sex ratios of each outcome during the risk and control periods for individuals aged ≥65 years In individuals aged ≥65 years, the sex ratios during the risk period within 10 days after vaccination and those during the control period from 11 to 180 days after vaccination and p-values are shown. Outcomes in ≥20 cases were tested using Fisher's exact test. n/a, not available.

Outcome	Risk period			Control period			p-value
	Male	Female	Sex ratio	Male	Female	Sex ratio	
All-cause deaths	448	485	92	214	164	130	0.0050
Ischemic heart diseases	46	44	105	16	13	123	0.83
Myocarditis/Pericarditis	3	7	43	3	7	43	1.00
Cardiac arrhythmias	10	4	250	4	0	n/a	
Aortic aneurysm/dissection	9	31	29	3	8	38	0.71
Heart failure	34	42	81	5	11	45	0.41
Intracerebral hemorrhage	15	15	100	12	6	200	0.37
Subarachnoid hemorrhage	5	12	42	1	2	50	1.00
Cerebral infarction	6	12	50	12	13	92	0.37
Respiratory failure	5	6	83	4	2	200	
Interstitial lung diseases	4	2	200	12	5	240	1.00
Pulmonary embolism	2	5	40	2	3	67	
Pneumonia	17	12	142	20	6	333	0.17
Sepsis	8	10	80	5	7	71	1.00
Anaphylaxis	7	5	140	0	0	n/a	
Thrombocytopenia	1	2	50	6	3	200	
Aspiration pneumonia/Asphyxia	24	31	77	12	5	240	0.094
Marasmus	10	21	48	1	1	100	1.00
Unexplained Deaths	92	88	105	39	20	195	0.051

In Group 2, the sex ratio for all-cause deaths during the risk period was 204, which was significantly higher than the 111 observed during the control period (p=0.044). Regarding the outcomes in ≥20 cases, the sex ratio during the risk period was higher than that during the control period for conditions including ischemic heart disease, subarachnoid hemorrhage, and unexplained deaths. However, no significant differences were observed between the sex ratios by period. The sex ratio during the risk period for myocarditis/pericarditis was 800, eight times higher than that during the control period. However, the number of cases was small (17 cases). For unexplained deaths, autopsies were performed in nine of the 51 cases. The statistical power of the tests for all-cause deaths was 0.50. The sex ratios and p-values for each outcome are summarized in Table 2.

**Table 2:** Sex ratios of each outcome during the risk and control periods for individuals aged ≤64 years In individuals aged ≤64 years, the sex ratios during the risk period within 10 days after vaccination and those during the control period from 11 to 180 days after vaccination and p-values are shown. Outcomes in ≥20 cases were tested using Fisher's exact test. n/a, not available.

Outcome	Risk period			Control period			p-value
	Male	Female	Sex ratio	Male	Female	Sex ratio	
All-cause deaths	116	57	204	39	35	111	0.044
Ischemic heart diseases	15	4	375	5	3	167	0.63
Myocarditis/Pericarditis	8	1	800	4	4	100	
Cardiac arrhythmias	15	4	375	5	0	n/a	0.54
Aortic aneurysm/dissection	2	3	67	3	1	300	
Heart failure	9	2	450	1	1	100	
Intracerebral hemorrhage	6	3	200	2	0	n/a	
Subarachnoid hemorrhage	5	9	56	1	5	20	0.61
Cerebral infarction	1	1	100	0	1	0	
Respiratory failure	0	1	0	0	1	0	
Interstitial lung diseases	0	0	n/a	0	1	0	
Pulmonary embolism	0	1	0	1	4	25	
Pneumonia	0	1	0	1	0	n/a	
Sepsis	2	2	100	0	0	n/a	
Anaphylaxis	0	0	n/a	0	0	n/a	
Thrombocytopenia	0	0	n/a	0	0	n/a	
Aspiration pneumonia/Asphyxia	1	1	100	0	1	0	
Marasmus	0	0	n/a	0	0	n/a	
Unexplained Deaths	28	9	311	8	6	133	0.30

## Discussion

The present analysis showed that the sex ratio for all-cause deaths during the risk period was significantly lower than that during the control period in Group 1 (Table 1); conversely, it was significantly higher than that during the control period in Group 2 (Table 2). If there is no effect on the occurrence of death, there should be no difference in sex ratios by period. Thus, this finding indicates that vaccination may influence the occurrence of death during the risk period and might be associated with death. The power of the test in Group 1 was 0.79, whereas it was low at 0.50 in Group 2. The test power is thought to have been low because of the small sample size in Group 2. Thus, the result in Group 2 should be carefully interpreted.

In Group 1, regarding the outcomes in ≥20 cases, the sex ratios during the risk period were lower than those during the control period for conditions including ischemic heart disease, aortic aneurysm/dissection, intracerebral hemorrhage, subarachnoid hemorrhage, cerebral infarction, interstitial lung diseases, pneumonia, aspiration pneumonia/asphyxia, marasmus, and unexplained deaths. The accumulation of these outcomes may have resulted in a lower sex ratio during the risk period for all-cause deaths. As Yamashita et al. indicated in 2022, the reasons for the low sex ratios are believed to be that women's body weight in Japan is generally lower than that in the USA and European countries [4] and that women's immune responses to vaccines are stronger than those of men [7]. No significant differences were observed between the sex ratios by period across any of the outcomes. One contributing factor for this is thought to be the limited number of cases for each outcome.

In Group 2, regarding the outcomes in ≥20 cases, the sex ratios during the risk period were higher than those during the control period for conditions including ischemic heart disease, subarachnoid hemorrhage, and unexplained deaths. No significant differences were observed between the sex ratios by period across any of the outcomes. This is thought to be due, in part, to the small number of cases for each outcome. The sex ratio for myocarditis/pericarditis during the risk period was 800, which was eight times higher than that during the control period. However, the number of cases was small (17 cases). Since autopsies were performed in only nine of the 51 cases of unexplained deaths, some myocarditis/pericarditis cases may be included within the unexplained deaths category. Myocarditis is a complication of vaccination, especially in young adults and adolescent males [8]. One contributing factor for the high sex ratio of all-cause deaths during the risk period is thought to be the high number of myocarditis/pericarditis deaths including undiagnosed cases.

In a cohort study in Japan published in 2022, there was no significant increase in all-cause mortality owing to vaccination [1]. This does not contradict the results of the present study. When the mortality rate of vaccination is exceptionally low, even if there is an association, no significant difference may be found in the cohort study. The results of a cohort study provide support for vaccine safety. However, this does not indicate that vaccine-related deaths are nonexistent; it only indicates that their number is not large enough to make a significant difference. Japan has relief services for adverse health effects that provide financial support to patients when it is evaluated as undeniable that the patient died from the vaccination [9]. On this occasion, it is difficult to determine whether a post-vaccination death is incidental or vaccine-related. The manual "Causality assessment of an adverse event following immunization" published by the World Health Organization describes it as follows: At the individual level, the scientific basis for the criteria in the causality assessment process includes six pieces of evidence, and population-based evidence for causality is among them [10]. Thus, when a cohort study does not detect a significant difference due to a low mortality rate, further statistical analysis is necessary to determine whether to provide financial support. Vaccines are administered to all people, including healthy individuals. Thus, vaccines require a higher level of safety than pharmaceuticals used for treatment and should have an exceptionally low vaccination mortality rate. Therefore, it is necessary to analyze vaccine safety with statistical methods that can detect significant differences even when the vaccination mortality rate is exceptionally low.

Another limitation of this cohort study [1] is that study data were adjusted for the Charlson comorbidity index [11] based on the diagnoses made using Japanese administrative claims data. The index predicts mortality by classifying or weighting comorbid conditions. This claims data included diagnoses but not the disease severities. To determine the disease severities, it is necessary to examine the medical records. Thus, the disease severity bias between the vaccinated and unvaccinated groups may not have been properly corrected. The MHLW recommends not vaccinating patients when the disease is worsening or when the patient is generally debilitated [12]. Thus, the proportion of those conditions in the unvaccinated group may be higher than that in the vaccinated group. The study base of the SCCS is restricted to the individuals who experience a particular outcome [13]. These individuals include both vaccinated and unvaccinated groups [14], and the incidence rate of adverse events during the risk period is compared with that in the control period. Therefore, this bias can influence the results of all-cause deaths in a cohort study and an SCCS.

In an SCCS in Japan, there was no significant increase in all-cause mortality owing to vaccination [1]. This may not also contradict the results of the present study. There are two possible reasons for this. The first is the disease severity bias, and the second is the longer risk period, which was set within 21 days after vaccination instead of 10 days. In the present study, post-vaccination deaths were concentrated within 10 days after vaccination. Therefore, the results may have been different if the risk period was set within 10 days.

The method of comparing the incidence rate of adverse events in the vaccinated group during the risk period with that in the control period is called the self-controlled risk interval design (SCRI). Baker et al. described seven study designs for monitoring the association between vaccinations and adverse events, and the SCRI is among them [14]. This method has the unique feature of implicitly controlling for time-invariant confounding factors and has been used in multiple studies for vaccine safety [14-16]. Furthermore, this method has the advantage of being unaffected by the disease severity bias as the study base is restricted to only vaccinated groups. In this respect, the SCRI is superior to cohort studies and the SCCS when it is difficult to correct the disease severity bias. The SCCS may be advantageous when the outcome is rare [14,15]. The author believes that the SCRI may have the same advantage. Yamashita et al. stated in their paper, “we evaluated if the death reports would show the uneven distribution after vaccination” [4]. To the author's knowledge, the SCRI and the method of comparing sex ratios have not been discussed in terms of incidentality or even distribution. The author believes that the method of comparing sex ratios by period stems from the SCRI and that both methods aim to determine whether adverse events are incidental or not. Specifically, they assess an even distribution in the vaccinated group rather than comparing the incidence rate of the vaccinated group to that of the unvaccinated group. Notably, a significant difference may be detected using these methods even in cases where the vaccination mortality rate is exceptionally low, which distinguishes them from cohort studies. The American Statistical Association statement asserted that a scientific conclusion should not be determined by a single p-value alone and that an analysis using multiple methods is recommended [17]. Therefore, the author contends that when a significant difference is not identified in a cohort study due to a low mortality rate of vaccination, further analysis should be undertaken using either an SCRI or a comparison of sex ratios by period.

The percentage of reported deaths during the risk period was 71% in Group 1 and 70% in Group 2 (Figures 1, 2), indicating that the mortality rate during the risk period may have been higher than that during the control period. However, it is not appropriate to conclude that there is an association between vaccination and deaths after vaccination. This is due to the existence of a reporting bias. In Japan, physicians have reported only cases in which they suspected that the vaccination was associated with death. Thus, in these cases, the greater the number of days from vaccination to death, the less likely the cases were to be reported by physicians. Consequently, this bias could have led to a higher mortality rate in the reported cases during the risk period compared with that in the control period. In contrast, the author believes that comparing sex ratios by period is less prone to reporting bias. This is because the decision to report by a physician is often not influenced by the sex of the patient. Therefore, this method may be useful for analyzing data with reporting bias. The author believes that comparing sex ratios may not provide conclusive evidence, but it can offer valuable insights into assessing vaccine safety, especially when the mortality rate of vaccination is exceptionally low.

In addition to comparing sex ratios, an SCRI analysis is required to evaluate vaccine safety. In analyzing the SCRI, it is necessary to collect data to eliminate reporting bias as much as possible. However, the Japanese MHLW did not collect such data. Therefore, data collection methods in Japan should be reviewed. It is desirable to establish a system in Japan equivalent to the Vaccine Safety Datalink in the United States.

This study has some limitations. First, the number of days from vaccination to death may vary depending on treatment. Second, this study did not consider the effects of the vaccination after 11 days. Third, there may have been a few physicians who used sex as a determining factor in reporting. Fourth, the mortality rates could not be calculated because the analysis was performed only for deaths after vaccination. Fifthly, the test result for all-cause deaths in Group 2 should be carefully interpreted as the statistical power was 0.50. Sixthly, the findings of outcomes with small sample sizes should be carefully interpreted. Finally, the analysis results should be carefully interpreted because not all deaths reported to the MHLW were related to vaccination. Incidental deaths may be included in the reported deaths.

## Conclusions

The present analysis showed significant differences between sex ratios during the risk and control periods for individuals aged ≥65 years and ≤64 years in the reported deaths after BNT162b2 mRNA vaccination in Japan. This finding indicates that the vaccination may influence the occurrence of death during the risk period and might be associated with death. In a cohort study in Japan, there was no significant increase in all-cause mortality owing to vaccination. This does not contradict the results of the present study. The results of a cohort study provide support for vaccine safety. However, this does not indicate that vaccine-related deaths are nonexistent; it only indicates that their number is not large enough to make a significant difference. An SCRI and a comparison of sex ratios by period may be useful in examining the association between vaccination and deaths after vaccination when a cohort study does not detect a significant difference due to a low mortality rate. The latter approach may be particularly useful for analyzing data with reporting bias. The author believes that this approach may not provide conclusive evidence, but it can offer valuable insights into assessing vaccine safety.
